# Chromosomal and molecular evidence for presence of Polyommatus (Agrodiaetus) poseidon (Lepidoptera, Lycaenidae) in Caucasus region

**DOI:** 10.3897/CompCytogen.v9i2.5020

**Published:** 2015-06-03

**Authors:** Vladimir A. Lukhtanov, Valentin V. Tikhonov

**Affiliations:** 1 Department of Karyosystematics, Zoological Institute of Russian Academy of Sciences, Universitetskaya nab. 1, 199034 St. Petersburg, Russia; 2 Department of Entomology, Faculty of Biology, St. Petersburg State University, Universitetskaya nab. 7/9, 199034 St. Petersburg, Russia; 3 North Caucasus Federal University, Pushkin str., 1, Stavropol 355009 Russia

**Keywords:** *Agrodiaetus*, chromosome number, *COI*, karyotype, Lycaenidae, *Polyommatus*

## Abstract

We show how combination of chromosomal and molecular markers can be applied for proper species identification in *Agrodiaetus* Hübner, 1822 blue butterflies. Using this approach we provide first evidence for presence of Polyommatus (Agrodiaetus) poseidon (Herrich-Schäffer, [1851]) in Georgia.

## Introduction

The blue butterfly subgenus *Agrodiaetus* Hübner, 1822 belongs to the genus *Polyommatus* Latreille, 1804 (Talavera et al. 2013). In the last years, this group become a model system for study of speciation and chromosome evolution (Lukhtanov et al. 2015, Vershinina et al. 2015). Despite this, its taxonomy is still poorly elaborated and identification of individual species is difficult due to their morphological similarity. Species within the subgenus are mostly uniform and exhibit few differences in characters traditionally used in classification, such as wing pattern and/or aspects of the male and female genitalia (Lukhtanov et al. 2006, Vila et al. 2010). The genus was estimated to have originated very recently (Kandul et al. 2004) and, thus, many *Agrodiaetus* species may have not had sufficient time to acquire extensive genetic differences. In particular, *COI* barcode gap is low or even absent between numerous closely related species of Polyommatus (Agrodiaetus) (Wiemers and Fiedler 2007). In opposite to majority of other butterflies and moths (Lukhtanov 2014), many *Agrodiaetus* species have evolved distinctive karyotypes. They show one of the highest interspecific karyotypic diversities known in the animal kingdom with haploid chromosome numbers ranging from n = 10 to n = 134 (Lukhtanov et al. 2005). Therefore, karyotypic features provide important identification characters for many described species that are virtually indistinguishable by their morphology. However, it should be noted that in few cases the chromosome number may be identical in different species (see Results and Discussion).

Here we show how combination of chromosomal and molecular markers can be applied for proper species identification in *Agrodiaetus*. Using this approach we provide first evidence for presence of Polyommatus (Agrodiaetus) poseidon (Herrich-Schäffer, [1851]) in Georgia.

## Material and methods

The samples used for molecular and chromosomal analysis were collected in Georgia (Akhaltsikhe, 41.60N, 43.06E, 1000 m alt., 18 July 2014, V. Lukhtanov et V. Tikhonov leg., samples 2014VL56, 2014VL57, 2014VL58, 2014VL62, 2014VL63, 2014VL64, 2014VL65, 2014VL68, 2014VL69, 2014VL70). The methods of DNA sequencing, chromosomal analysis and phylogenetic inference were described previously (Lukhtanov and Dantchenko 2002a, Lukhtanov et al. 2008, 2014, Vershinina and Lukhtanov 2010, Przybyłowicz et al. 2014). Additional samples of *Polyommatus* belonging to Polyommatus (Agrodiaetus) poseidon species complex (Kandul et al. 2007) were used for comparison.

## Results and discussion

The species Polyommatus (Agrodiaetus) poseidon (= Lycaena poseidon var. mesopotamica Staudinger, 1892, synonymized with *Polyommatus poseidon* by Schurian et. 1992) is known to be an endemic of the Middle East sporadically distributed from Kütahya in West Turkey to Artvin in North-East Turkey (Hesselbarth et al. 1995). Phenotypically similar, but chromosomally distinct species Polyommatus (Agrodiaetus) putnami (Lukhtanov & Dantchenko, 2002) was described from East Turkey (provinces Erzurum and Ağri) (Lukhtanov and Dantchenko 2002b). The last taxon is allopatric in distribution with Polyommatus (Agrodiaetus) poseidon and differs from *Polyommatus poseidon* by chromosome number and karyotype structure (Lukhtanov and Dantchenko 2002b). Polyommatus (Agrodiaetus) poseidon has relatively low haploid chromosome number (from n=19 on the south and east of the distributional range to n=21 in the north), all the chromosomes form a gradient size row with no especially large or small chromosomes (de Lesse 1963, Kandul and Lukhtanov 1997). Chromosome numbers n=22 and n=23 were also found in the northern population as intraindividual occasional deviations from the basic n=21 (de Lesse 1963). Polyommatus (Agrodiaetus) putnami has higher chromosome numbers (from n=24 to n=27, with n=26 as a distinct mode). Its karyotype is asymmetrical and includes chromosomes of two distinct classes: class of large chromosomes and class of small chromosomes (Lukhtanov and Dantchenko 2002b). Currently Polyommatus (Agrodiaetus) putnami is treated as a distinct species (Lukhtanov and Dantchenko 2002b, Wiemers 2003, Wiemers and Fiedler 2007) or a subspecies of Polyommatus (Agrodiaetus) poseidon (Tshikolovets 2011).

The taxon Polyommatus (Agrodiaetus) deebi (Larsen, 1974) discovered in Lebanon and Syria is often considered as a subspecies of Polyommatus (Agrodiaetus) poseidon (e.g. Tshikolovets 2011), however, it differs in chromosome number (n=17, Larsen 1975) and may represent a different species (Eckweiler and Häuser 1997). The taxon Polyommatus (Agrodiaetus) damocles krymaeus (Sheljuzhko, 1928) was also considered as subspecies of Polyommatus (Agrodiaetus) poseidon (Hesselbarth et al. 1995), however, with respect to mitochondrial genes *COI* and *COII* it is very distant from Polyommatus (Agrodiaetus) poseidon and was shown to be a subspecies of Polyommatus (Agrodiaetus) damocles (Herrich-Schäffer, [1844]) (Lukhtanov et al. 2005, Kandul et al. 2007).

Males of Polyommatus (Agrodiaetus) poseidon have plesiomorphic (Kandul et al. 2004, Lukhtanov et al. 2005) blue colouration of the upper side of the wings with no specific morphological characters. Therefore their morphological discrimination from phenotypically similar Polyommatus (Agrodiaetus) caeruleus (Staudinger, 1871), Polyommatus (Agrodiaetus) damocles and Polyommatus (Agrodiaetus) damonides (Staudinger, 1899) is difficult. With respect to *COI* barcodes, Polyommatus (Agrodiaetus) poseidon is indistinguishable from Polyommatus (Agrodiaetus) hopfferi (Herrich-Schäffer, [1851]) and Polyommatus (Agrodiaetus) putnami (Wiemers & Fiedler, 2007). As it was stated above, the chromosome number varies within Polyommatus (Agrodiaetus) poseidon (de Lesse 1963, Kandul and Lukhtanov 1997, Lukhtanov and Dantchenko 2002b) and thus overlap with chromosome numbers found in Polyommatus (Agrodiaetus) elbursicus (Forster, 1956), Polyommatus (Agrodiaetus) cyaneus (Staudinger, 1899), Polyommatus (Agrodiaetus) ectabanensis (de Lessse, 1963), Polyommatus (Agrodiaetus) hamadanensis (de Lesse, 1959), Polyommatus (Agrodiaetus) alcestis (Zerny, 1932), Polyommatus (Agrodiaetus) altivagans (Forster, 1956), Polyommatus (Agrodiaetus) mithridates (Staudinger, 1878), Polyommatus (Agrodiaetus) shirkuhensis ten Hagen et Eckweiler, 2001 and Polyommatus (Agrodiaetus) pierceae (Lukhtanov & Dantchenko, 2002) (Kandul et al. 2007, Lukhtanov et al. 2014).

**Figure 1.**
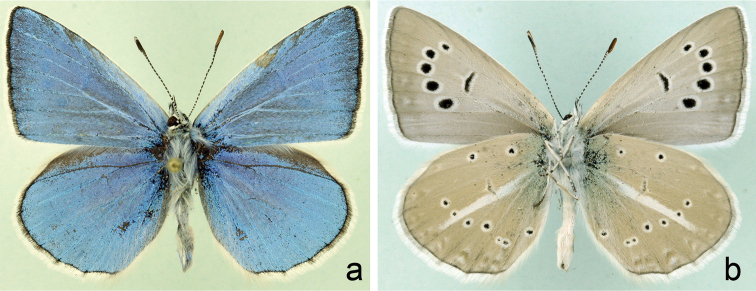
Polyommatus (Agrodiaetus) poseidon from Akhaltsikhe, Georgia. **a** male, upperside **b** male, underside.

**Figure 2.**
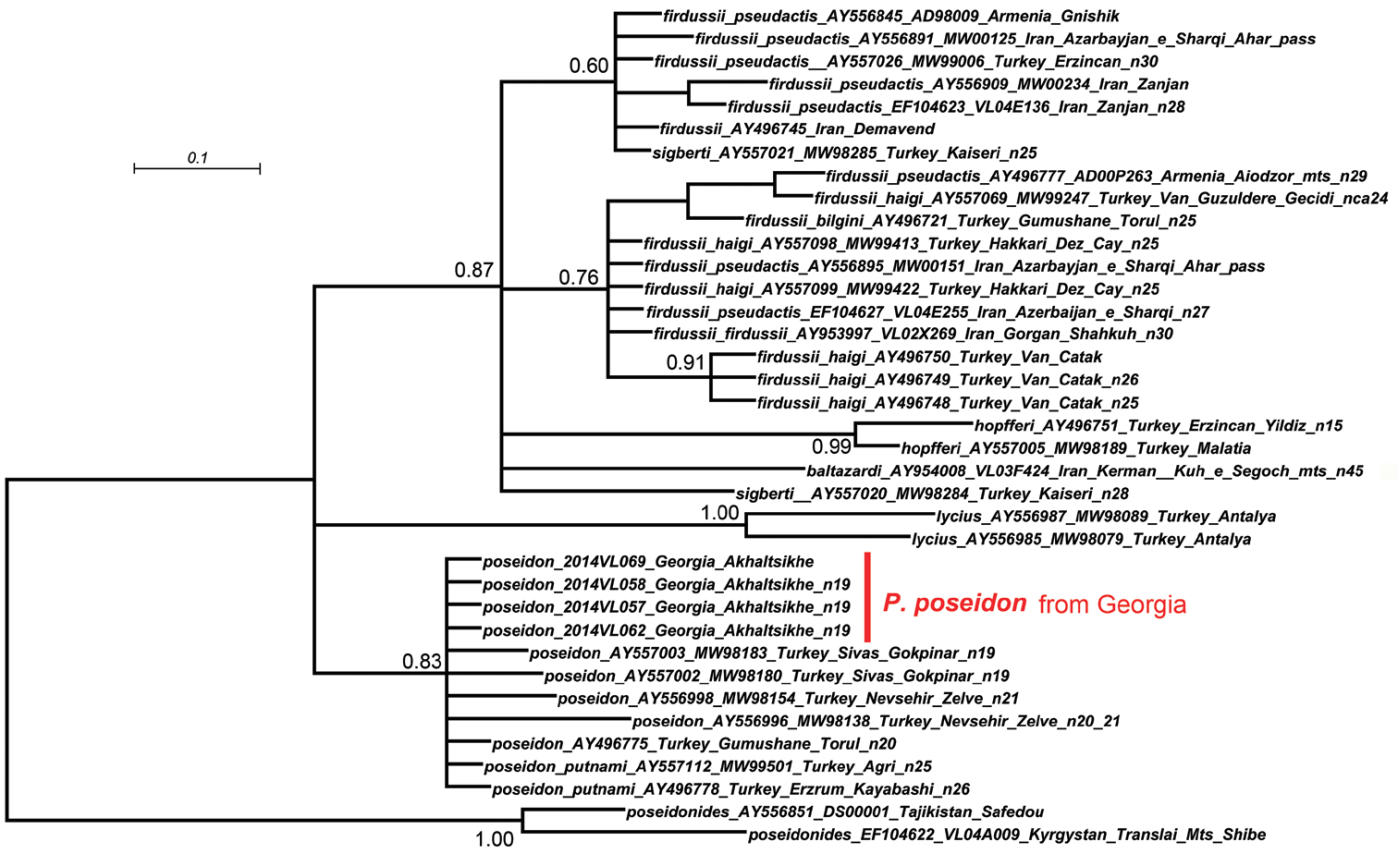
Bayesian tree of the species close to Polyommatus (Agrodiaetus) poseidon inferred from *COI* sequences. Posterior probability values >50% are shown.

A population of blue butterflies which were morphologically similar to Polyommatus (Agrodiaetus) poseidon (Fig. 1) was discovered near Akhaltsikhe in Georgia in 2013 by V.Tikhonov and I. Kostyuk. In 2014 the locality was visited again in order to collect material available for molecular and chromosomal study. Molecular analysis of this material revealed that *COI* barcodes were completely identical or nearly identical (barcode gap from 0 to 0.6%) in population from Akhaltsikhe and other populations of Polyommatus (Agrodiaetus) poseidon and Polyommatus (Agrodiaetus) putnami (Fig. 2).

**Figure 3.**
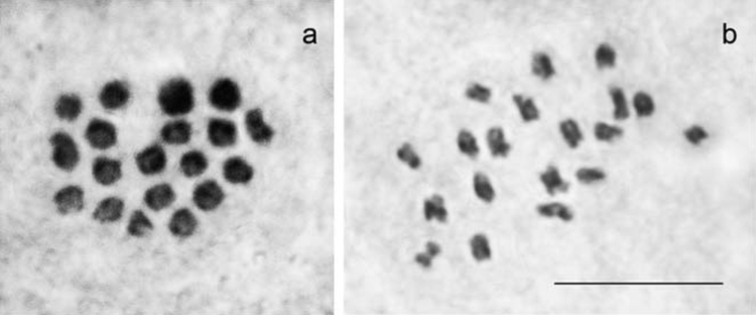
Male karyotype of Polyommatus (Agrodiaetus) poseidon from Georgia. **a** sample 2014VL57, metaphase I, n = 19 **b** sample 2014VL62, metaphase II, n = 19. Bar = 10 μm.

The haploid chromosome number n=19 was found in MI and MII cells of three studied individuals (2014VL57, 2014VL58, 2014VL62) (Fig. 3). All chromosome elements formed a gradient size row. The karyotype contained no exceptionally large or small chromosomes. In this respect, the population from Akhaltstikhe is indistinguishable from populations of Polyommatus (Agrodiaetus) poseidon from Amasya (de Lesse 1963) and Artvin (Kandul and Lukhtanov 1997), but differs from Polyommatus (Agrodiaetus) putnami (n=26) (Lukhtanov and Dantchenko 2002b).

Thus, although in the studied case neither the DNA barcodes nor chromosomal numbers are species-specific characters, their combination clearly indicates that the population from Akhaltsikhe should be identified as Polyommatus (Agrodiaetus) poseidon. This is the first evidence of Polyommatus (Agrodiaetus) poseidon for Georgia and for Caucasus region at whole.
